# Analog Memory and Synaptic Plasticity in an InGaZnO-Based Memristor by Modifying Intrinsic Oxygen Vacancies

**DOI:** 10.3390/ma16247510

**Published:** 2023-12-05

**Authors:** Chandreswar Mahata, Hyojin So, Soomin Kim, Sungjun Kim, Seongjae Cho

**Affiliations:** 1 Division of Electronics and Electrical Engineering, Dongguk University, Seoul 04620, Republic of Korea; chandreswar@gmail.com (C.M.);; 2 Department of Electronic and Electrical Engineering, Ewha Womans University, Seoul 03760, Republic of Korea

**Keywords:** InGaZnO, charge trapping, multilevel memory, short-term plasticity, paired-pulse facilitation, spike-rate-dependent plasticity, long-term potentiation

## Abstract

This study focuses on InGaZnO-based synaptic devices fabricated using reactive radiofrequency sputtering deposition with highly uniform and reliable multilevel memory states. Electron trapping and trap generation behaviors were examined based on current compliance adjustments and constant voltage stressing on the ITO/InGaZnO/ITO memristor. Using O_2_ + N_2_ plasma treatment resulted in stable and consistent cycle-to-cycle memory switching with an average memory window of ~95.3. Multilevel resistance states ranging from 0.68 to 140.7 kΩ were achieved by controlling the V_RESET_ within the range of −1.4 to −1.8 V. The modulation of synaptic weight for short-term plasticity was simulated by applying voltage pulses with increasing amplitudes after the formation of a weak conductive filament. To emulate several synaptic behaviors in InGaZnO-based memristors, variations in the pulse interval were used for paired-pulse facilitation and pulse frequency-dependent spike rate-dependent plasticity. Long-term potentiation and depression are also observed after strong conductive filaments form at higher current compliance in the switching layer. Hence, the ITO/InGaZnO/ITO memristor holds promise for high-performance synaptic device applications.

## 1. Introduction

Resistive switching memories, functioning as synaptic devices with analog switching features, have gained significant attention owing to their low-operational voltage, high speed, and uncomplicated sandwiched structural characteristics. These make them promising candidates for the next generation of nonvolatile memory solutions [1,2,3]. Following the electroformation of conductive filaments (CFs) in the presence of an external electric field, the generation and disruption of CFs near the electrodes determine the high-resistance (OFF) and low-resistance (ON) states. Nevertheless, achieving a stable and gradual change in conductance, which is contingent on the controlled migration of oxygen vacancies, poses a challenge when memristors are incorporated to simulate synaptic functions. Transparent resistive random access memories (RRAMs) have also recently gained attention for their potential use in invisible memory and display applications [4]. Numerous efforts have been directed toward transparent RRAMs based on zinc oxide and doped zinc oxide, primarily because of their broadband gap, high transparency, the ability for large area deposition, and compatibility with the back-end of line devices in the complementary metal-oxide semiconductor process [5,6,7,8,9]. Indium and gallium-doped zinc oxide (InGaZnO) has been identified as a promising compound for switching in the field of RRAMs owing to its high-transparent properties and low-temperature fabrication process [10,11]. However, several challenges must be addressed for the practical application of InGaZnO-based memristors in synaptic devices, including issues such as cycle-to-cycle variability and the gradual transition between multiple memory states. The level of oxygen content in InGaZnO films was found to play a significant role in influencing the formation of oxygen vacancies (V_O_) during the electroforming of CFs. Improvement of multilevel memory state and gradual resistive switching have been reported in the context of a bilayer-based structure involving binary oxides and InGaZnO. In this setup, control over CFs was achieved at the interface, which led to more dependable resistive switching characteristics [9,12,13,14,15]. The proper introduction of metal ion doping in InGaZnO has also shown a high On/Off ratio along with enhanced resistive switching properties [11]. Plasma treatment during the deposition of InGaZnO has been explored as a means to enhance switching uniformity. This is achieved by regulating the oxygen vacancies through redox reactions during the formation and disruption of CFs [8,9,16].

In this study, the resistive switching layer, InGaZnO, was deposited using reactive radiofrequency (RF) sputtering. It is important to highlight that the controlled manipulation of oxygen vacancies based on O_2_ + N_2_ plasma treatment is the critical factor influencing the gradual resistive switching observed in the indium-doped tin oxide (ITO)/InGaZnO/ITO-based memristor. The consistent presence of stable resistive switching and synaptic properties demonstrate that controlled changes in conductance can be achieved by applying electrical presynaptic pulses. In this experiment, the memristor effectively emulates the behavior of a biological synapse, showcasing essential functionalities like spike amplitude-dependent plasticity (SADP), short-term plasticity (STP), long-term plasticity (LTP), paired-pulse facilitation (PPF), and spike rate-dependent plasticity (SRDP). The ITO/InGaZnO/ITO memristor, following O_2_ + N_2_ plasma treatment, exhibits a gradual alteration in conductance when subjected to both direct current (DC) and pulse voltages, thus making it a promising candidate for use in synaptic devices.

## 2. Materials and Methods

The initial substrate used for the RRAM fabrication process was a commercially available ITO conductive layer on a glass substrate with a resistivity in the range of 70–100 Ω/sq. Before depositing the switching layer for memristor fabrication, the bottom ITO electrode was cleaned using acetone, isopropyl alcohol, and DI water in an ultrasonic bath. Subsequently, a thin film of InGaZnO with a thickness of ~20 nm was deposited using RF magnetron sputtering using a commercial target with an elemental composition ratio of In_2_O_3_:Ga_2_O_3_:ZnO = 1:1:1. During the deposition, the RF power was set to 120 W under a base pressure of 3 mTorr. Two separate sets of switching layers were deposited using the mixed ambient atmosphere with an Ar:O_2_:N_2_ ratio of 10:5:5 sccm and an Ar:N_2_ ratio of 10:10 sccm. Photolithography was employed to define the top electrode to create square array devices; this resulted in an area of 100 × 100 µm^2^. Finally, the top electrodes were patterned based on a lift-off process following the RF-sputtered ITO deposition, which used a power of 60 W and a gas flow of 8 sccm Ar. All DC and synaptic properties of ITO/InGaZnO/ITO memristors were assessed using the Keithley 4200-SCS semiconductor parameter analyzer (Keithley Instruments in Cleveland, OH, USA) and 4225-PMU pulse module with a manual probe station (MS-TECH). The bottom ITO electrode was connected to the ground, while electrical bias was applied to the top ITO electrodes during DC resistive switching and pulse synaptic measurements.

## 3. Results and Discussion

### 3.1. Charge Trapping and Soft-Breakdown Characteristics of ITO/InGaZnO/ITO Memristor

**Figure 1:**
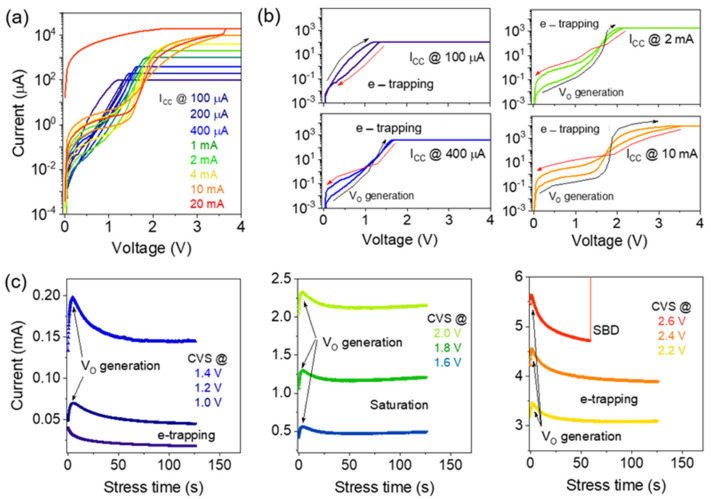
(**a**,**b**) Current–voltage characteristics were obtained for the indium–doped tin oxide (ITO)/Indium and gallium–doped zinc oxide (InGaZnO)/ITO memristor with O_2_ + N_2_ plasma treatment by increasing the current compliance limit from 100 µA to 20 mA. The right panel highlights the charge–trapping behavior at the specific I_CC_ values of 0.1, 0.4, 2, and 10 mA. (**c**) Current vs. time plots during the 120 s constant voltage stressing confirm the electron trapping and oxygen vacancy generation at different times and stress voltages ranging from 1.4 to 2.6 V.

The charge trapping and generation of new extrinsic defects were studied at increasing current compliance and constant voltage stressing (CVS) on the ITO/InGaZnO/ITO memristor, as shown in Figure 1a–c. The current compliance (I_CC_) varied from 100 µA to 20 mA to control the maximum voltage applied to the memristor in this experiment. Figure 1b presents four different stages of the current conduction in the O_2_ + N_2_ plasma-treated memristor. Using a positive bias at the top electrode under an I_CC_ of 100 µA, the fresh memristor shows predominantly electron trapping, where a decrease in current is observed. Increasing the I_CC_ at positive voltages eventually allows the use of higher voltages in the devices that generate oxygen vacancy defects (V_O_) in the switching layer. A soft breakdown was observed at an I_CC_ of 20 mA. At very high I_CC_ values, responses from electron trapping and new trap generation are observed. This phenomenon may be due to oxygen or nitrogen interstitials that generate electron traps [17]. Therefore, oxygen vacancies are generated in the memristor if the applied voltage is high enough and the CFs form slowly. Similar electron trapping and trap generation behaviors have been reported [18]. At low CVS values (1.0 V), electron trapping was confirmed from the current-time plots where the memristor currents continuously decrease, as shown in Figure 1c. Furthermore, by increasing the CVS amplitude from 1.2 to 2.4 V, both V_O_ generation and electron trapping were observed, as confirmed by the transient response of memristor currents. These results imply the generation of new electron trap states at higher CVS amplitudes that dominantly reduce the resultant currents, as shown in the right panel of Figure 1c.

### 3.2. Gradual Bipolar Resistive Switching of ITO/InGaZnO/ITO Memristor

**Figure 2:**
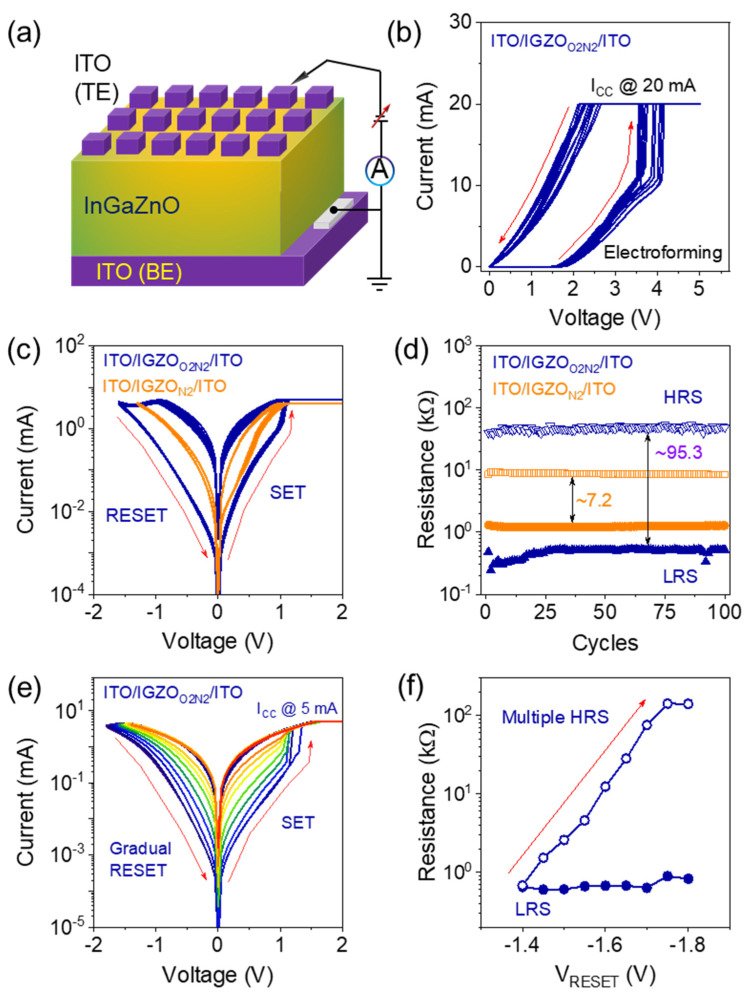
(**a**) Schematic of the cross-sectional structure of ITO/InGaZnO/ITO memristors with a bias applied to the top electrode. (**b**) Forming process of the O_2_ + N_2_ plasma-treated memristor without significant variation. (**c**) Bipolar resistive switching comparison of N_2_ and O_2_ + N_2_ plasma-treated ITO/InGaZnO/ITO memristors. (**d**) The direct current endurance characteristics of both memristors show a stable I_on_/I_off_ ratio up to 100 cycles. (**e**,**f**) Gradual RESET characteristics and increment of high-resistance state (HRS) attained by increasing V_RESET_ from −1.4 to −1.8 V at the current compliance of 5 mA with a step of −0.05 V.

The schematic of the structure of the ITO/InGaZnO/ITO memristor is shown in Figure 2a, where the InGaZnO switching layer is sandwiched between the top ITO (biased) and bottom (grounded) electrodes. The CFs are formed by applying a positive bias at a top electrode at a current compliance of 20 mA, as clearly shown in Figure 2b. Multiple O_2_ + N_2_ plasma-treated memristor forming processes demonstrated an average electroforming voltage of 3.8 V without any significant variation in pristine (before electroforming) device resistance. During the electroforming process, oxygen vacancies (V_O_) are generated, and oxygen ions (O^2−^) are moved to the electrode interface and act as the oxygen reservoir layer due to the application of the external bias at the top electrode [19]. Typical bipolar resistive switching characteristics of ITO/InGaZnO/ITO memristor devices showing 100 DC switching cycles after O_2_ + N_2_ and N_2_ plasma treatment are plotted in Figure 2c. In the case of the N_2_ plasma-treated InGaZnO-based memristor, the initial current (without electroforming) was higher than that following O_2_ + N_2_ plasma treatment, and spontaneous current relaxation was observed owing to the weak filament formation. Compared with the switching behavior reported by Zhang et al. [9], single-layer O_2_ + N_2_ plasma-treated memristors in this experiment demonstrate stable and reliable cycle-to-cycle memory switching responses. The distribution of high- and low-resistance states (HRS/LRS) is compared in Figure 2d; findings show a stable I_on_/I_off_ ratio for 100 DC cycles. The memory window increased from ~7.2 to 95.3 for N_2_ and O_2_ + N_2_ plasma-treated ITO/InGaZnO/ITO memristors, which indicates the advantage of O_2_ incorporation and reduction in oxygen vacancies inside the InGaZnO switching layer [8,16]. Therefore, the oxygen vacancy density can be reduced significantly by using O_2_ plasma during the sputtering process, thus improving the memory-switching behavior of InGaZnO-based memristors. In addition to bipolar switching, gradual changes in high-resistance states are emulated by stepwise increasing (indicated by red arrow) the reset voltage (V_RESET_) from −1.4 to −1.8 V (−0.05 V step), as shown in Figure 2e, where the SET I_CC_ was kept at 5 mA. In the gradual RESET process, O^2^ at the ITO/InGaZnO interface (oxygen reservoir) moves back near the CFs and recombines with V_O,_ eventually rupturing the CFs in a stepwise manner and yielding multiple HRSs [9,20]. Multilevel resistance states from 0.68 to 140.7 kΩ are obtained at the read voltage of 0.2 V and plotted in Figure 2f. Compared with the previous report, stable and nonvolatile multilevel resistance states of the ITO/InGaZnO/ITO memristor are achieved with O_2_ + N_2_ plasma treatment in this experiment, which exhibits excellent outcomes in artificial synaptic device applications.

### 3.3. Pulse Amplitude-Dependent Short-Term and Long-Term Synaptic Weight Modulation

**Figure 3:**
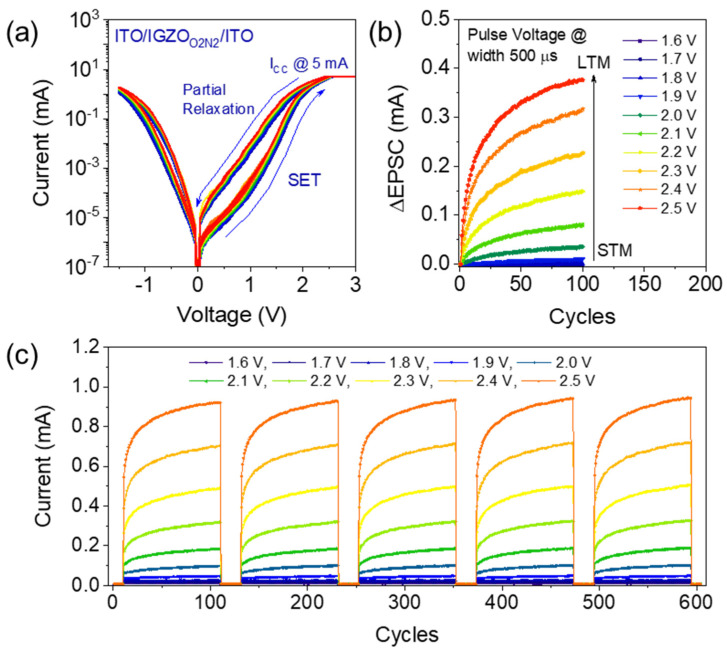
(**a**) Relaxation I–V behavior of ITO/InGaZnO/ITO memristors without electroforming. Partial relaxation behavior was observed in the reverse bias condition. (**b**) Potentiation behaviors following the application of 100 identical pulse sequences at increasing amplitudes from 1.6 to 2.5 V with a pulse width of 500 µs. The excitatory postsynaptic current (EPSC) obtained in the ITO/InGaZnO/ITO memristor confirms the transition from short- to long-term plasticity by increasing the pulse amplitude. (**c**) The reliability of the memory transition behavior was confirmed by measuring postsynaptic currents at increasing amplitudes (from 1.6 to 2.5 V) using five consecutive potentiation cycles.

Short-term memory behavior has been examined in the ITO/InGaZnO/ITO memristor with O_2_ + N_2_ plasma treatment by applying a lower current compliance level without any electroforming (strong filament formation), as shown in Figure 3a. When subjected to this reduced current compliance, volatile and relatively weak CFs were formed inside the switching layers owing to low-density oxygen vacancy generation. Therefore, owing to the volatile nature of the weak filaments, the resistance of the memristor partially recovered to its initial HRS during the reverse sweep of applied positive voltages. The partial relaxation occurred near the ITO/InGaZnO owing to the redox reaction that led to the recombination of V_O_ and O^2−^ [21]. Furthermore, a complete RESET was achieved after a small negative DC sweep voltage (0–−1.5 V) was applied, as confirmed in Figure 3a. This transient modification of synaptic weight (observed in terms of short-term plasticity) plays a vital role in mimicking pattern high-pass filtering and the learning mechanisms associated with biological brain functions. Short-term learning rules can be emulated by controlling the postsynaptic currents depending on the low- and high-presynaptic pulse voltage amplitudes [22]. In Figure 3b, the nonlinear generation of excitatory postsynaptic current (EPSC) is presented after the application of a 100-pulse sequence with increasing pulse amplitudes from 1.6 to 2.5 V and a pulse width of 500 µs, which demonstrates the dependable occurrence of short-term and long-term memory functions. At lower pulse amplitudes (ranging from 1.6 to 2.0 V), redox reactions will likely take place easily owing to the dominant oxygen reaction with V_O_. However, after applying a threshold pulse voltage (V_th_) amplitude of ≥2.1 V, a transition of the memory state of the synaptic device from STP to LTP was observed. For voltages lower than V_th_, the ΔEPSC was found to be lower than 0.034 mA; by contrast, for voltages higher than V_th_, the EPSC increased from 0.08 to 0.38 mA. The transition occurred owing to the accumulation of a notably higher number of oxygen vacancies at the switching layer interface compared with the recombination of oxygen ions, which led to a significant increase in the EPSC. Therefore, the aforementioned synaptic current emulation in artificial ITO/InGaZnO/ITO synapses with short- to long-term memory transitions is in good agreement with biological memory function. Cycle-to-cycle reliability was further confirmed with five consecutive sets of potentiation behaviors by increasing the pulse amplitude from 1.6 to 2.5 V, as shown in Figure 3c.

### 3.4. PPF and Pulse Frequency-Dependent Synaptic Properties

**Figure 4:**
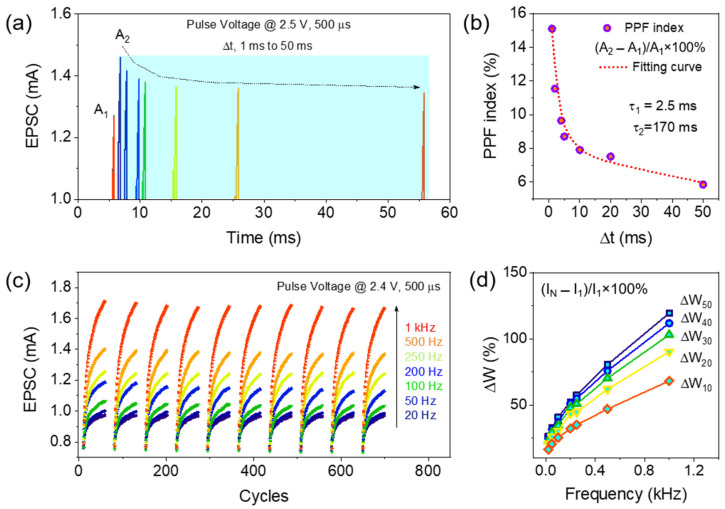
(**a**) Postsynaptic current is triggered by applying paired pulses with an amplitude of 2.5 V and a duration of 500 µs at different intervals (from 1 ms to 50 ms) on the ITO/InGaZnO/ITO memristors. (**b**) The experimental paired-pulse facilitation index was calculated as a function of pulse interval and plotted as (A_2_ − A_1_)/A_1_ × 100% along with a simulated curve using Equation (1). (**c**) Obtained EPSC from the ITO/InGaZnO/ITO synapse, varying pulse frequency from 20 Hz to 1 kHz with an amplitude of 2.4 V and a duration of 500 µs for 50 consecutive presynaptic spikes. (**d**) The synaptic weight change was calculated as (I_N_ − I_1_)/I_1_ × 100% at different frequencies and spike numbers.

Synaptic weight modifications depend on the spike rate or interval between two intermediate presynaptic stimuli, which is an important STP response in biological memory function [23]. The change in the EPSC increment depends on the spike amplitude and width of the stimuli that preceded the response. In this section, the dynamic synaptic plasticity of the ITO/InGaZnO/ITO memristor is studied based on the PPF process. The EPSC was measured at two adjacent closed presynaptic pulses (with amplitudes and widths of 2.5 V and 500 µs, respectively) applied at different time delays (Δt) (between 1 ms and 50 ms), as shown in Figure 4a. Similar to the biological synapse, the peak EPSC values are increased by the second pulse, which is larger than the first presynaptic spike [24]. At the lowest presynaptic pulse time delay (1 ms), the EPSC increased from 1.26 to 1.45 mA, whereas by increasing the Δt to 50 ms, the EPSC achieved was 1.33 mA at the second pulse. This phenomenon confirms the learning rules in this memristor, according to which faster repetition of training pulses enhances the synaptic weight like that in a biological system. At the positive pulse, V_O_ and O^2−^ were separated, and the V_O_s accumulated promptly near the weak CFs. At elongated interspike interval times, partial V_O_ was recombined with O^2−^ which drifted back to the switching layer; these events caused the CFs to rupture, and consequently, the conductance of the memristor decreased, thus indicating that the forgetting operations were related to the STP. Although the recombination rate is affected by the shorter time interval and the partial residual V_O_ near the weak CFs substantially increases the resultant EPSC at the second pulse, a higher PPF index was obtained at the shorter paired pulse interval. The PPF index, defined by (A_2_ − A_1_)/A_1_ × 100%, was calculated and plotted in Figure 4b, where A_1_ and A_2_ are the EPSC peak amplitudes obtained at the first and second pulses [25]. An exponential PPF index decay from 15.1% to 5.8% was observed by increasing Δt from 1 to 50 ms, which is analogous to biological memory [26]. The experimental PPF obtained was well fitted with the double-exponential decay function (indicated by the red line) in Figure 4b, according to

PPF% = C_1_ exp(−Δt/τ_1_) + C_2_ exp(−Δt/τ_2_) (1)

where the time constants τ_1_ and τ_2_ are the characteristic relaxation times for fast and slow decay conditions, Δt is the wait time between two successive presynaptic spikes, and C_1_ and C_2_ are the initial PPF magnitudes [24]. Relaxation time constants τ_1_ and τ_2_ extracted from the simulations using the above equation were 2.5 and 170 ms, respectively. Therefore, the short-term PPF synaptic function was successfully mimicked in the ITO/InGaZnO/ITO memristor.

Additionally, SRDP related to the dynamic filtering for transmission of information was also mimicked in the ITO/InGaZnO/ITO synapse, where EPSC depended on the presynaptic pulse frequency, as shown in Figure 4c [27]. The increasing amplitude of EPSC was observed from 0.96 mA to 1.7 mA by increasing the spike frequency from 20 Hz to 1 kHz, thus indicating the characteristics of high-pass filtering biological memory function. The EPSC increment was due to the reduced pulse interval, which eventually hindered the recombination of V_O_ with O^2−^. The synaptic weight gain calculated as (I_N_ − I_1_)/I_1_ × 100% at different frequencies is plotted in Figure 4d, where I_N_ is the measured EPSC at the end of 10, 20, 30, 40, and 50 successively applied presynaptic spikes. Synaptic weight gain was gradually enhanced with increasing stimulus frequencies and pulse numbers. The maximum weight gain at a pulse frequency of 1 kHz varied from 68.2% to 119.4% after the application of 10 to 50 presynaptic spikes, respectively. Therefore, in both PPF and SRDP, synaptic emulations of the V_O_ generation process using positive voltage pulses modulated the conductance of the ITO/InGaZnO/ITO artificial synapse temporarily. After the application of the first pulse, owing to the low density of the generated V_O_, it tended to recombine with O^2−^ ions after the pulse (with a high-pulse interval) was withdrawn. However, if the immediate next pulse interval is very short, the residual V_O_ augments the EPSC at the subsequent pulse, which further enhances the synaptic weight, which gradually depends on the pulse number and higher frequencies, as presented in Figure 4c,d.

### 3.5. Pulse Amplitude and Width-Dependent Long-Term Potentiation Properties

**Figure 5:**
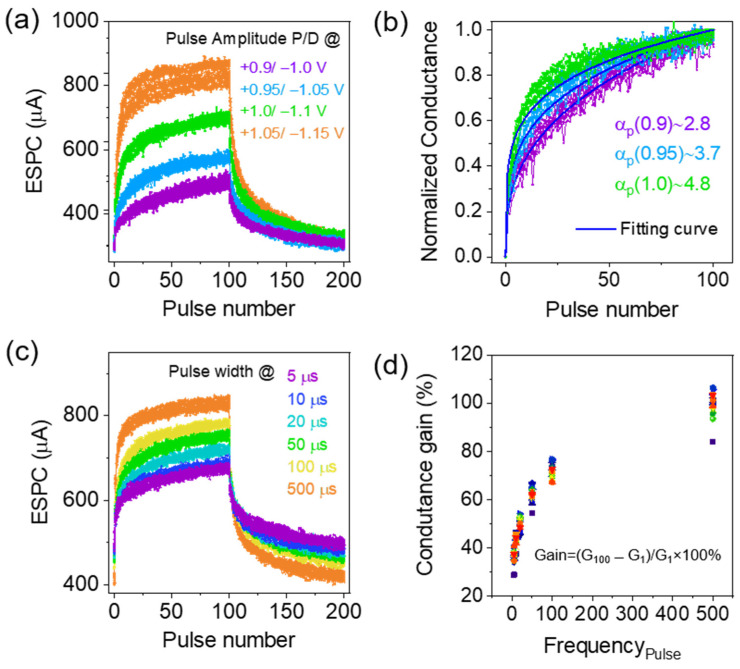
(**a**) Potentiation and depression properties of ITO/InGaZnO/ITO memristor with O_2_ + N_2_ plasma treatment after electroforming using varying pulse amplitudes (from 0.9 to 1.05 V and −1.0 to −1.15 V, respectively). (**b**) Gradual normalized conductance at different amplitude pulse trains is fitted using Equation (2). (**c**) Postsynaptic current modulations obtained by varying the pulse width from 5 to 500 µs and (**d**) change in conductance gain at increasing presynaptic pulse widths.

Emulation of biological synaptic behavior implemented in this section with applications of potentiation and depression presynaptic pulses resulted in LTP and long-term depression (LTD) [28,29]. Increasing (identical) pulse amplitudes of 100 presynaptic pulses were applied to the top electrodes from 0.9 to 1.05 V and −1.0 to −1.15 V (pulse width = 200 µs, read pulse amplitude = 0.5 V) to attain gradual EPSC increases and decreases, as shown in Figure 5a. The dynamic current responses in Figure 5a clearly show the pulse amplitude dependence, where the postsynaptic current increases/decreases and becomes higher and faster at larger positive/negative pulse amplitudes. Reliable cycle-to-cycle variability for 20 LTP/LTD cycles achieved in this experiment proved to be suitable for synaptic device applications. Therefore, the SADP was successfully emulated in the ITO/InGaZnO/ITO memristor, which is consistent with the SET/RESET voltages found in the DC switching characteristics [30]. By increasing the pulse amplitude at the presynapse, the accumulation of V_O_ near the CFs was significantly increased, depending on the potentiation pulse amplitudes. At the negative depression pulses, the mobile O^2−^ was easily recombined with the V_O,_ and synaptic conductance decreased. Therefore, ITO/InGaZnO/ITO successfully demonstrated the capacity to mimic dynamic biological synaptic weight functions in terms of LTP and LTD. Similar synaptic functions were emulated in several previous studies by increasing pulse amplitudes [31,32,33,34]. The potentiation conductance at different pulse amplitudes was normalized and plotted in Figure 5b, and Equation (2) was then applied for fitting.

(2) $$G={\left((G^{\alpha }{}_{LRS\;}-G^{\alpha }{}_{HRS\;})\times w+G^{\alpha }{}_{HRS\;}\right)}^{1/\alpha }$$

In this equation, *G_LRS_* and *G_HRS_* represent the conductance states in the low and high resistance states, respectively. The parameter “*w*” varies from 0 to 1, and “α” represents the nonlinearity factor [35,36]. The nonlinearity factor increased from 2.8 to 4.8 when the pulse voltage amplitudes changed from 0.9 to 1.0 V, similar to the prediction described elsewhere [34]. Potentiation and depression behavior under increasing pulse width from 5 to 500 µs with fixed pulse amplitudes of 1 V/−1.1 V are demonstrated in Figure 5c. The increasing EPSC behavior depends on the pulse width at the potentiation process and confirms the migration of oxygen vacancies on the CFs, thus leading to increasing conductance rates. Extended pulse widths provide increasing periods to allow the accumulation of oxygen vacancies; this process further controls the EPSC increment. Steady 20-cycle-to-cycle increases/decreases of EPSCs at positive and negative 100-pulse spike trains are consistent, which demonstrates that the memristor is excellent for artificial synaptic device applications. In Figure 5d, the conductance gain (G_100_ − G_1_)/G_1_ × 100% is plotted as a function of the presynaptic pulse width. Improved cycle-to-cycle variability of G_gain_% and precise control of CFs in the ITO/InGaZnO/ITO memristor are promising for dynamic conductance changes [33].

We conducted a thorough analysis and comparison of our InGaZnO-based memristive device’s performance against previously reported memristive devices. In this section, we present a summary of the analog switching characteristics of our InGaZnO-based memristive device following O_2_ + N_2_ plasma treatment, as outlined in Table 1. Leveraging a comprehensive literature survey, we compared the detailed bipolar resistive and synaptic characteristics of various InGaZnO-based memristive devices. Our findings reveal that our InGaZnO-based memristive device demonstrates promising multilevel switching characteristics with a controlled change in HRSs. Notably, our device exhibits highly concentrated set and reset operations in comparison to bilayer and plasma-treated memristive devices. Based on these results, we posit that mixed O_2_ + N_2_ plasma-treated InGaZnO-based memristive devices hold significant potential for future applications in high-density memory and neuromorphic computing. The exceptional performance and controllability of our device render it a promising candidate for advancing these fields.

## 4. Conclusions

In summary, the application of O_2_ + N_2_ plasma treatment to the InGaZnO switching layer effectively enabled the modulation of oxygen vacancy concentration and allowed the control of multilevel memory states governed by V_RESET_. The initial leakage current in the ITO/InGaZnO/ITO memristor was reduced following a combined O_2_ + N_2_ plasma treatment (compared with N_2_ plasma treatment). This reduction was primarily ascribed to the diminished presence of oxygen vacancies within the switching layer. Following the 0O_2_ + N_2_ plasma treatment, an average electroforming voltage (approximately equal to 3.8 V) was observed, accompanied by a stable Ion/Ioff ratio of 95.3 in ITO/InGaZnO/ITO memristor cases. Multiple HRSs were observed, which ranged from 0.68 to 140.7 kΩ; these were achieved by systematically controlling the V_RESET_ with a linear increase. The transition from short- to long-term memory states was successfully achieved by applying a threshold voltage amplitude of ≥2.1 V, thus demonstrating reliable cycle-to-cycle variability. STP characteristics were effectively emulated through PPF and spike frequency-dependent synaptic weight gain in ITO/InGaZnO/ITO memristors. The accumulation of oxygen vacancies near the conductive filaments was significantly enhanced by increasing the pulse amplitude and width, thereby influencing the synaptic conductance during LTP and LTD. The multilevel conductance characteristics and dependable synaptic behavior suggest that the ITO/InGaZnO/ITO memristor holds promise for future, large-scale manufacturing applications.

## Figures and Tables

**Table 1:** Performance comparison of InGaZnO-based memristive devices.

Device Structure	SET/RESET (V)	Endurance Cycles	Memory Window	Multilevel Switching	Switching Behaviors	Synaptic Properties	Reference
Ag/a-IGZO/TiO_2_/ ITO	+1.5/−1.5	250	<10	No	Analog	No	[5]
ITO/IGZO/ ZrO_x_/Ti	−2.4/+2.3	10^3^	~100	No	Analog	No	[6]
ITO/IGZO/ ITO	+4.0/−3.8	50	~10	No	Digital	No	[8]
Pt/IGZO/ IGZO:N/TiN	+0.75/−0.75	100	~100	No	Analog	No	[9]
Pt/a-IGZO/Pt	+1.6/−1.0	100	~10	No	Digital	No	[10]
Ag/IGZO (Ru)/Pt	+0.25/−0.6	50	~10^4^	No	Digital	No	[11]
Mo/Al_2_O_3_/ IGZO/Pd	+5.1/−4.8	500	>10	No	Analog	No	[14]
Ti/TaOx/ IGZO/Pt	+0.75/−0.9	50	10^3^	Yes	Analog	No	[15]
Al/IGZO/Al_2_O_3_/Al	+2.3/−0.6	100	10^4^		Digital	No	[16]
ITO/IGZO/ ITO	+1.0/−1.0	100	~10^2^	Yes	Analog	Yes	This study

## Data Availability

Data are contained within the article.
